# A novel peptide that inhibits E2F transcription and regresses prostate tumor xenografts

**DOI:** 10.18632/oncotarget.1809

**Published:** 2014-03-11

**Authors:** Xiaoqi Xie, Nitu Bansal, Tazeem Shaik, John E. Kerrigan, Tamara Minko, Olga Garbuzenko, Emine Ercikan Abali, Nadine Johnson-Farley, Debabrata Banerjee, Kathleen W. Scotto, Joseph R Bertino

**Affiliations:** ^1^ Rutgers Cancer Institute of New Jersey; ^2^ Department of Pharmaceutics Rutgers, the State University of New Jersey,Piscataway, New Jersey; ^3^ Departments of Pharmacology, Biochemistry, and Medicine,Robert Wood Johnson Medical School, Rutgers,The State University of New Jersey, New Brunswick, NJ

**Keywords:** prostate cancer, penetratin-peptide, Du-145 cells

## Abstract

E2F-1, a key transcription factor necessary for cell growth, DNA repair and differentiation, is an attractive target for development of useful anticancer drugs in tumors that are E2F “oncogene addicted”. A peptide, isolated from phage clones, based on its binding to an E2F-1 consensus sequence, was cytotoxic against a wide range of cancer cell lines.

The peptide was coupled to penetratin (PEP) and tested against prostate cancer cell lines. As the PEP was found to be relatively unstable in serum, it was encapsulated in PEGylated liposomes for in vivo studies.

The peptide was cytotoxic against prostate cell lines at low micromolar concentrations. Treatment of mice bearing the human Du-145 human prostate tumor with the PEP encapsulated in PEGylated liposomes (PL-PEP) caused tumor regression without significant toxicity.

The liposome encapsulated PEP has promise as an antitumor agent, alone or in combination with inhibitors of DNA synthesis.

## INTRODUCTION

While new anti-androgen therapies and new chemotherapeutic drugs have increased the survival of patients with castrate resistant prostate cancer, relapse eventually occurs and patients succumb to this disease. There continues to be a need for therapies with low toxicity in this population that may be used alone or in combination with currently approved treatments. In a previous publication we described studies of a peptide that inhibited transcription of E2F-1, and when coupled to a modified penetratin sequence to enhance uptake (PEP), and encapsulated in PEGylated liposomes (PL-PEP), inhibited growth of a human small cell carcinoma tumor in nude mice [1].

We targeted E2F, as the E2F family of transcription factors is critical to many cellular processes, including development, proliferation, DNA repair, and differentiation [2-6]. The retinoblastoma gene product (RB) is a critical player in the regulation of E2F. RB forms heterodimers with E2F-1, 2 and 3a, thereby suppresses E2F activity [7-9]. RB is phosphorylated by CDK 4, 6 and 2 in respnse to signals favoring cell cycle progression, and as a consequence, E2F is freed from this repressor complex, and transcribes many target genes. E2F-1 and E2F-3 are over expressed in many tumors and is associated with poor prognosis [10,11]. Therefore, targeting one or more activating E2Fs, has been recognized as an important and selective antitumor strategy. Several approaches have been described, that include oligonucleotide decoys to trap E2F-1, and generation of peptides that prevent the dimerization between E2F-1 and its DP partners [reviewed in 12, 13]. None to date have advanced to clinical trials for the treatment of cancer.

Herein we demonstrate that the PEP showed potent in vitro antitumor activity against prostate cancer cells and inhibition of tumor growth when xenografts of the castrate resistant cell tumor Du-145 were treated with the PEP encapsulated in PEGylated liposomes.

## RESULTS

### Cytotoxicity studies *in vitro*

The Du-145 cell line was the most sensitive of the prostate cancer cell lines to the PEP. Table I shows average IC_50_ values for a 24 h exposure and a 72 h exposure to daily administration of fresh drug. The IC_50_s were decreased with daily administration, as was also noted previously with the H-69 cell line [1], due to lack of stability in culture media with FBS. Our previous study showed that normal cell lines that included mesenchymal stem cells and hematopoetic stem cells from human marrow, and MEFs, were not affected by concentrations of 80 μM, the highest concentrations tested [1].

**Table 1:** Effect of the PEP on viability of prostate tumor cells Cells were treated with the PEP at various concentrations for either 24 h (first column) or fresh drug at the same concentation added daily x3 and then viability measured at 96 h (second column).

Cell Line	L-peptide 24h average IC_50_	L-peptide 3 days average IC_50_
Du-145	48 μM	30 μM
LnCaP	80 μM	45 μM
PC3	70 μM	75 μM

**Fig 1:**
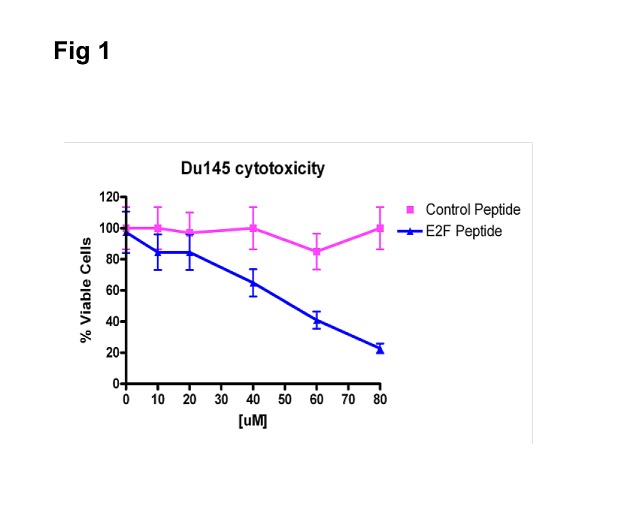
Cytotoxicity of PEP against Du-145 cells treated with various doses of PEP Control peptide has little effect on viability of Du-145 cells over the dose range tested.

We also tested a control PEP, with the 4 histidines replaced by glycines and compared this peptide to the lead PEP. As shown in Fig.1, the control peptide had little or no effect on cell growth when assayed against the Du-145 cell line.

### The PEP induces apoptosis

**Fig 2:**
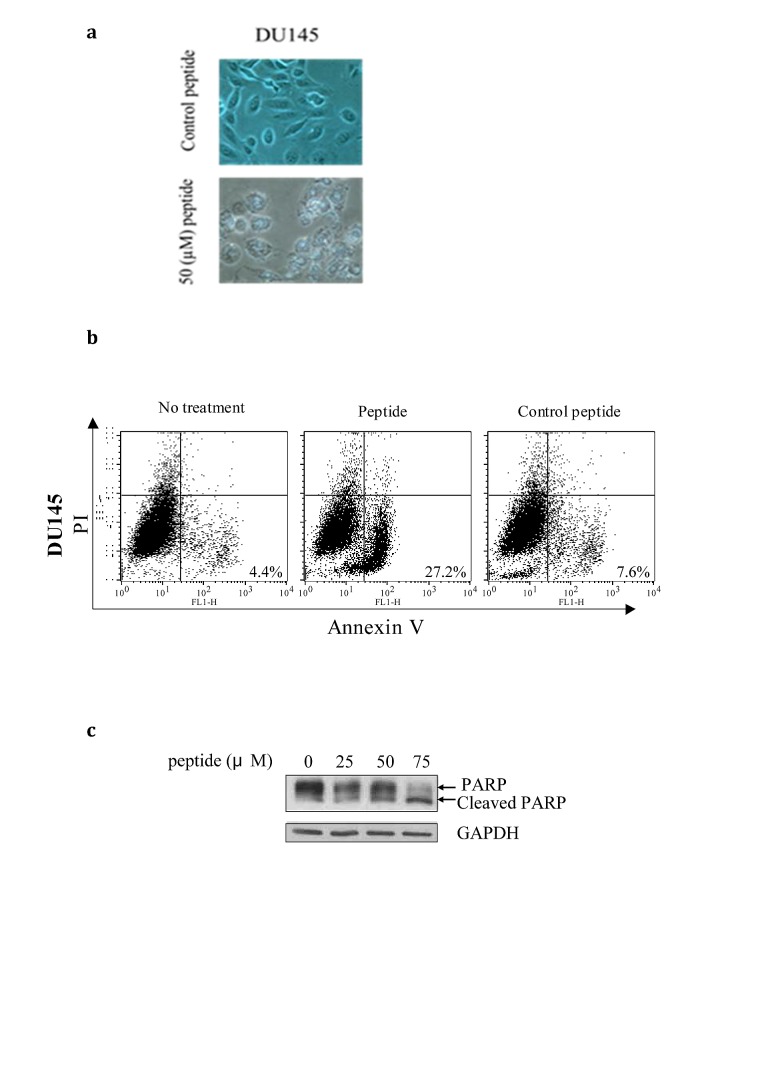
2a) The PEP induces morphological changes associated with apoptosis. Du-145 cells were treated with peptide and control peptide with an IC50 concentration for 6h and photographed.2b) apoptosis was analyzed for Annexin V and PI staining by flow cytometry (n=3). 2c) PEP treated Du- 145 showed PARP cleavage. Cells were treated for 24h at the indicated concentrations of PEP and analyzed by western blotting for PARP cleavage.

We observed that following a relatively short exposure of 6h to the PEP, Du-145 and LnCaP cells showed morphologic changes including loss of cell-cell contact and disintegration of the cellular and nuclear membrane indicating that the PEP was inducing apoptosis (Fig 2a). In order to confirm this, Du-145 cells, most sensitive to PEP, were treated with peptide for 6h at the IC50 concentration. Cells were analyzed after Annexin V and propidium iodide (PI) staining by flow cytometry. Annexin V staining alone indicates early apoptotic cells, while double staining by both Annexin V and PI indicates late apoptotic cells. Staining by PI alone indicates necrotic cells. As shown in Fig 2b, the percentage of apoptotic cells was significantly increased in the presence of the PEP, in Du-145 (from 7.6% to 27%). This observation was obtained in several other cancer cell types and PEP apoptosis was further confirmed by cleavage of apoptosis marker PARP (Fig.2c).

### Inhibitory effect of the PEP on the cell cycle

**Fig 3:**
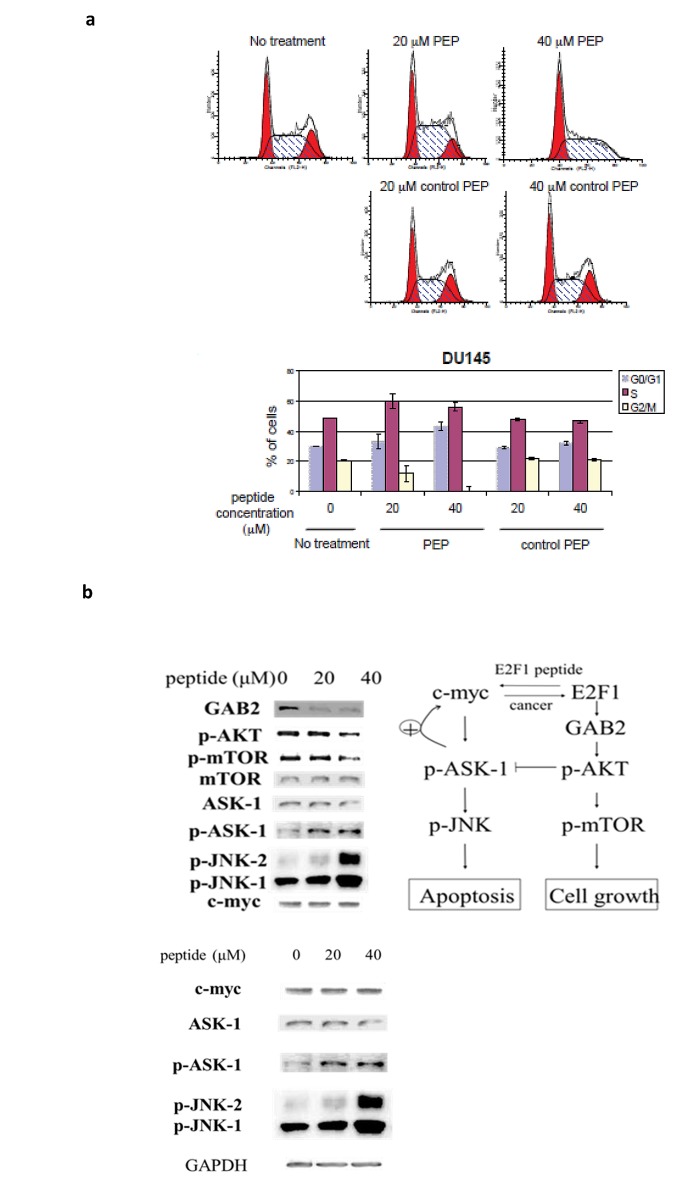
3a) Effect of PEP on the cell cycle: Following treatment of Du-145 cells with PEP for 12h and PI staining, cells were analyzed by flow cytometry and quantitated (bottom panel). 3b) Western analysis of proteins involved in the cell death response to the PEP. Cells were treated with 40 μM PEP for 24 h and proteins in the signal transduction pathway involving mTOR and JNK were analyzed.

Because E2F-1 plays an important role in cell cycle progression from G1 to S phase, we examined the effect of the peptide on the cell cycle of Du-145 cells. Cells were arrested in G1/G0 by incubation in serum free media, and after release into serum containing media the effect of the PEP and control PEP on the entrance of the cells into S phase was measured, using a concentration of the PEP that was slightly below the IC_50_. After overnight treatment, we observed that control PEP did not block cell progress from G1 to S phase, whereas PEP treated cells had an increase in the proportion of cells in G1/S of cell cycle with a corresponding decline in the population of G2/M phase cells (Fig 3a top and bottom panel). To determine if the PEP caused alterations in signal transduction pathways, DU145 cells were treated with 20 and 40 μM peptide overnight. The PEP activated the ASK1/JNK signaling pathway and inhibited the mTOR/PI3K pathway previously shown to be important [14] in apoptosis/cell growth induced by E2F-1 (Fig 3b).

### The PEP down regulates E2F-1 and downstream E2F targets

**Fig 4:**
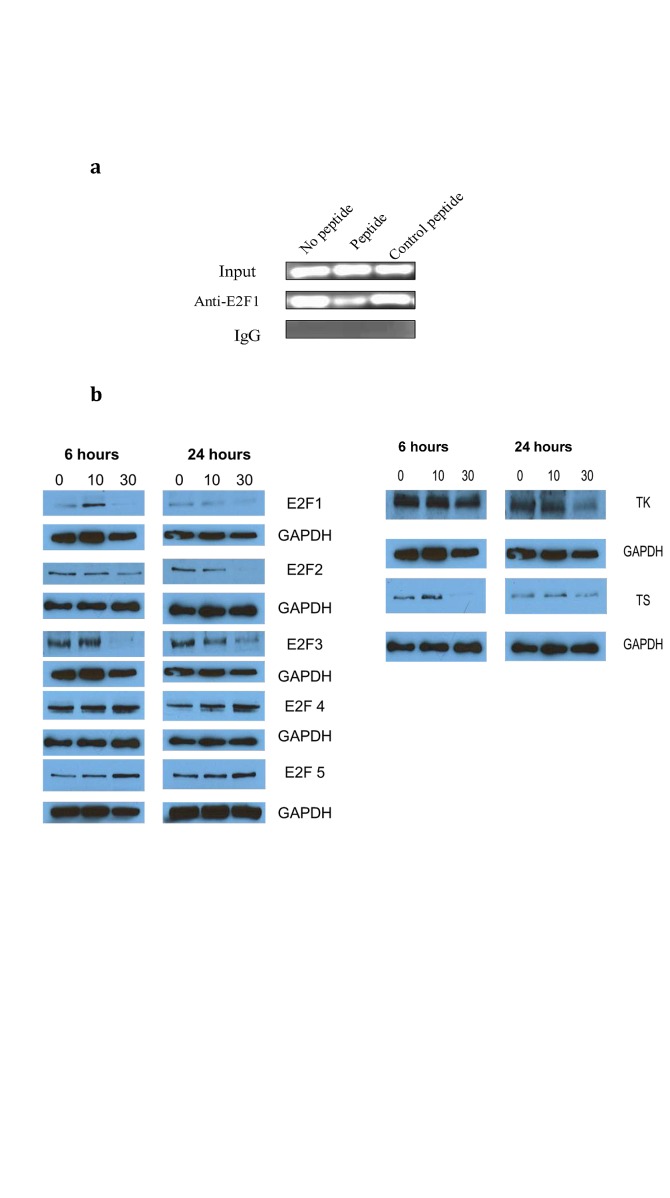
4a) The PEP inhibits E2F1 protein binding to its promoter. After serum-starved Du-145 cells were treated with E2F1 peptide and control peptide for 24 hours, a ChIP assay was performed with antibody against E2F1 and control IgG. The primers used in PCR flank the binding site in E2F promoter.4b) Effect of treatment of cells with the PEP on protein expression. Du-145 cells were treated with the PEP for 12h and protein analyzed for expression of target genes by Western blotting. Proteins analyzed included l E2F family members and E2F target proteins TS and TK.

In our previous publication we showed that the peptide inhibited the transcriptional activity of E2F1. To confirm these results in the Du-145 cell line we performed a ChIP assay to determine whether the peptide interfered with binding of E2F1 to its consensus DNA sequence. PEP treatment resulted in decreased PCR product following immunoprecipitation of E2F-1 bound DNA as compared to either control peptide treatment or no peptide treatment (Fig 4a). As E2F-1 transcription has been reported to regulate levels of enzymes essential for DNA synthesis, we measured the effect of the PEP on E2F-1 levels as well as certain enzymes required for synthesis of purines and pyrimidines. Fig 4b shows down regulation of E2F-1 protein, following PEP treatment of Du-145 cells. As expected, the protein levels as well as mRNA (data not shown) of its well defined downstream targets such as thymidylate synthase (TS), and thymidine kinase (TK), targets for clinically used anticancer drugs were also down regulated in Du-145 cells as the transcription of these mRNAs are regulated by E2F. Other targets such as dihydrofolate reductase (DHFR) and ribonucleotide reductase (RR) were also down regulated by the PEP (not shown).

### The combination of the PEP with MTX caused synergistic cell kill

**Fig 5:**
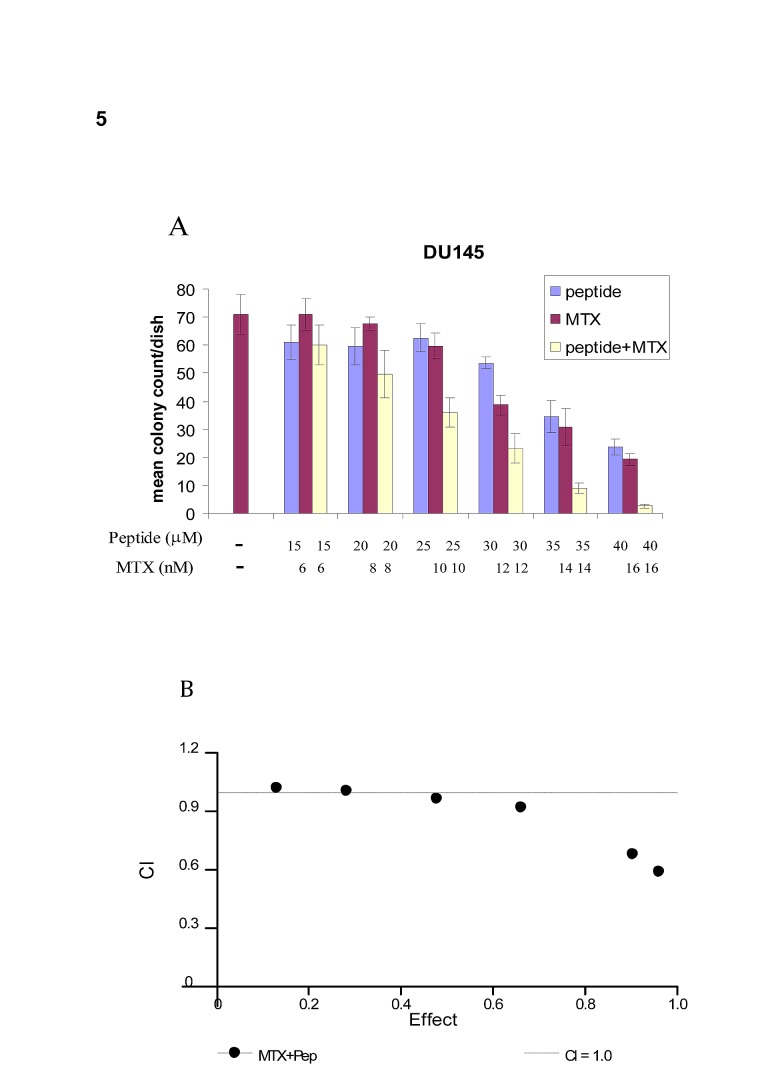
Chou-Talalay combination index analysis demonstrates synergism of the combination of PEP and MTX in Du-145 cells 200 Du-145cells were seeded in 6 well plates. PEP and MTX were added at various concentrations. After 2 weeks, colonies were counted and data shown in Fig 5a. CI values are shown in Fig5b.

Based on our observation that E2F peptide down-regulated certain target genes including DHFR, TS and TK we hypothesized that the peptide would synergize with inhibitors of these proteins required for S-Phase. As the peptide not only lowered levels of DHFR, but also thymidine kinase, thus also blocking the salvage pathway, we tested fixed ratios of the PEP with MTX, above and below the IC50 values for the individual drugs, and analyzed the results using the Chou-Talalay method of analysis to determine synergy, additive effects or antagonism [15]. At higher doses of MTX and PEP synergistic cell kill (CI values < 1.0) was observed as shown in Fig 5a and 5b.

### Xenograft studies

**Fig 6:**
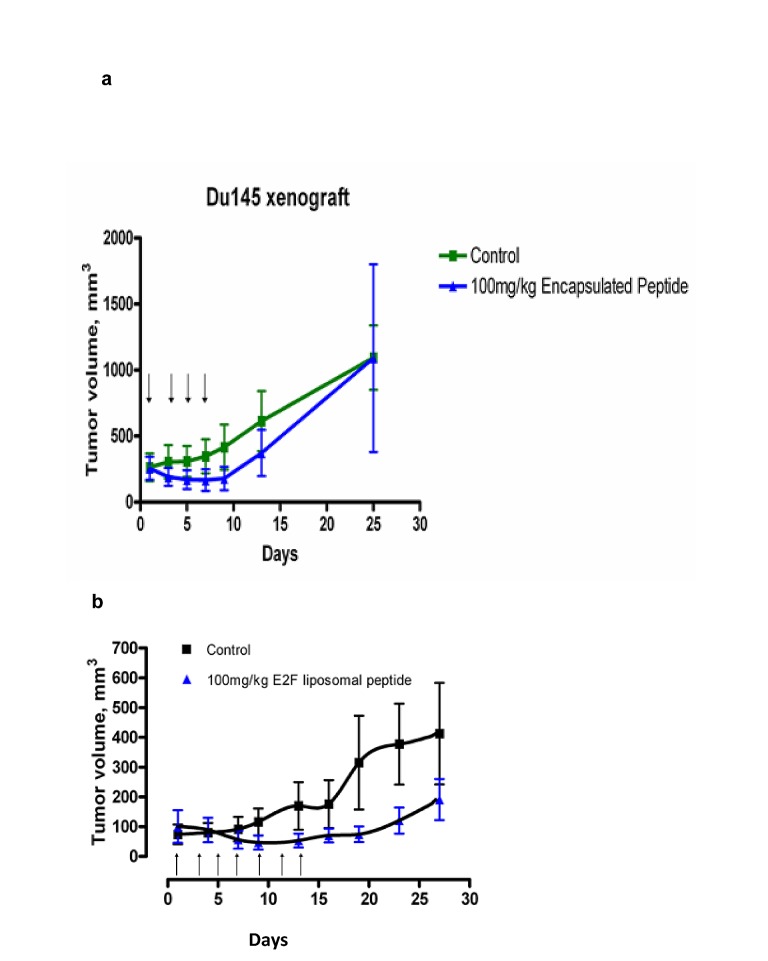
Xenograft studies Male mice (20-22 g) were inoculated subcutaneously with 10 million Du-145 cells and an equal volume of matrigel. When the tumors were palpable approximately 50-100 mm3, the animals were randomized into groups of 6 and either treated with 100 mg/kg of the PL-PEP, every other day for 4 doses (Fig 6a) or every other day for 7 doses ( Fig 6b). Tumor size and animal weight was measured every three days.

To increase the stability and tumor targeting of the PEP for xenograft studies in mice, we encapsulated the PEP in PEGylated liposomes (PL-PEP). We previously reported that The PL-PEP was rapidly taken up by H-69 and Du-145 tumor cells and entered the nucleus [1]. Xenografts of the Du-145 tumor cells were used to test the antitumor effects of the PL-PEP. Based on the MTD from the H-69 study, [1], the liposome encapsulated PEP, administered i.p @ 100 mg/Kg (0.2 ml) given every other day x 4, caused regression of these tumors without any observable toxicity (Fig 6a). As tumor growth recurred soon after treatment was stopped, in a second study, the PEGylated Pep was administered every other day @ 100 mg/ kg for 7 doses to determine if more frequent administration would prolong tumor growth inhibition and to assess toxicity (Fig 6b). This study showed that that tumor growth was inhibited as long as drug was administered, but again, when the PL-PEP was stopped, tumor growth resumed. No weight loss or toxicity was noted (data not shown). .

## DISCUSSION

The well-demonstrated association of E2F over expression (i.e. oncogene addiction) with maintenance of the malignant phenotype makes it an attractive target for new antitumor strategies. E2F-1 levels, as well as E2F-3 levels have been shown to be increased in several human cancers [16-21]. In some tumors over expression has been linked to amplification of the E2F-1 gene, which maps to 20q11.2 in the human genome [20]. Increased E2F-1 expression correlates with a poorer outcome in certain cancers such as lung cancer [11], and malignant melanoma [22]. A recent report also related over expression with melanoma progression and metastasis in a preclinical model [23]. Given the role of The E2F family of transcription factors in proliferation and tumor progression and metastasis, there have been several efforts to target one or more of the E2Fs as an antitumor strategy [reviewed in 12,13].

We reported previously that a peptide isolated by it's ability to bind tightly to an immobilized consensus E2F-1 sequence isolated from a phage display library down-regulated expression of not only E2F-1 but also E2F-2 and 4 [1]. When coupled to penetratin and modified further by replacing the methionine in the pentratin peptide by isoleucine, as this substitution allowed the PEP to be generated recombinantly (unpublished). The penetratin peptide (PEP) showed cytotoxic effects against several but not all human malignant cell lines and was not toxic at higher concentrations against normal human marrow hematopoietic and mesenchymal stem cells [1].

To enhance stability and the half -life of the PEP for in vivo studies we encapsulated the PEP into PEGylated liposomes. Regression of the H-69 SCLC xenografts, even when advanced resulted [1]. In this study we show that while the Du-145 cell line was less sensitive to the PEP as compared to the H-69 small cell cancer cell line, DU-145 ) xenografts also regressed when treated with the PL-PEP. Downstream effects of E2F inhibition resulted in down regulation of proteins that are targets for clinically useful chemotherapeutic drugs, including methotrexate (MTX, inhibitor of DHFR), the 5-fluoropyrimidines and pemetrexed (inhibitors of TS) and hydroxyurea (inhibitor of RR). Drugs that target these enzymes together with agents that lower E2F activity would be predicted to result in enhanced anti tumor effects. As an example of how this knowledge may lead to an effective drug combination with the PEP together with drugs that target these enzymes, we show that MTX and the PEP exhibit synergistic cell kill, likely due to decreased levels of DHFR and TK, the latter enzyme important for thymidine salvage.

Studies in progress will determine the effect of longer durations of treatment with the peptide, mechanisms of resistance and combinations of the peptide with agents used to treat prostate cancer, that include taxotere and DNA damaging agents, given the role of E2F-1 in DNA repair [5].

## MATERIALS AND METHODS

### Cell culture

Prostate cancer cell lines LnCaP, PC3, and Du-145 was obtained from ATCC and cultured in RPMI medium supplemented with 10% fetal bovine serum, penicillin, and streptomycin

### Cell viability assay

Human prostate cancer cells were plated in 24 well plates and treated with the PEP and control PEP respectively at various concentrations for 24 hours. The percentage of viable cells was determined by trypan blue staining and counting in a Vicell counter.

### Reverse transcription-PCR

Du-145 cells were treated with PEP for 12 h. RNA was isolated using TRIzol reagent (Invitrogen, Carlsbad, CA). RT-PCR was performed with the following primer pairs:

R2, 5′-TGGAGGATGAGCCGCTGCTGAGA-3′and 5′-TTGACACAAGGCATCGTTTCAATGG-3′;

E2F1, 5′-AGGCTGGACCTGGAAACTGACCAT-3′ and 5′-AGCTGCGTAGTACAGATATTCATCA-3′;

TS, 5′-GCGCTACAGCCTGAGAGATGAATT-3′ and 5′-CTTCTGTCGTCAGGGTTGGTTTTG-3′;

TK1,5′-GCATTAACCTGCCCACTGTGCTGC-3′ and 5′-GTGCCGAGCCTCTTGGTATAGGC-3′.

DHFR 5′-TAAACTGCATCGTCGCTGTGT-3′, and 5′-AGGTTGTGGTCATTCTCTGGAAA-3′

### Flow cytometry analysis

Cell cycle analysis: PEP or control PEP treated cells were fixed with cold ethanol, stained with PI/Rnase staining buffer (BD Biosciences, San Jose, CA) and analyzed by flow cytometry FACScan (Becton Dickinson, Franklin Lakes, NJ). All analyses were performed in triplicate.

### Apoptosis assay

Apoptosis were performed with Annexin V-FITC apoptosis detection kit 1 according to the manufacturer's instructions (BD Biosciences, San Jose, CA). All analyses were performed in triplicate.

### Chromatin immunoprecipitaion assay

ChIP was carried out as described previously [1].

### Immunoblotting Analysis

Cells were treated with indicated concentrations of peptide for 6 or 24h, harvested and lysates prepared for

### Western blot analysis

SDS-PAGE electrophoresis and Western blotting was performed according to standard procedures with 30 μg of whole-cell extracts. Antibodies used were: anti-E2F1 (KH95 Santa Cruz); anti-TK (3B3.E11 Santa Cruz); anti-R2 (I-15 Santa Cruz); anti-TS and anti DHFR antibody; Anti-Parp (Ab-2 Oncogene Science, Cambridge, MA).

### Clonogenic assay

Du-145 cells were seeded in six-well plates at 200 cells per well. The following day, the medium was replaced with medium containing PEP and MTX at various concentrations. After 2 weeks, colonies were stained with Crystal Violet and individual colonies counted.

### Pegylated liposomal encapsulation of PEP

PEGylated liposomes were prepared as previously described [1].

### Xenograft Studies

Du-145 cells (5x10^6^ cells per mouse) were injected into the flanks of nude mice and tumors allowed to develop. When tumors became palpable (100-200 cumm), tumor bearing animals were randomized into control and treatment groups and treated i.p. with doses of the PEP or empty PEGylated liposomes. Tumor volumes were measured serially using the formula (axb^2^/2) and expressed in mm^3^.
